# Effect of Mechanical Stimuli on the Phenotypic Plasticity of Induced Pluripotent Stem-Cell-Derived Vascular Smooth Muscle Cells in a 3D Hydrogel

**DOI:** 10.1021/acsabm.3c00840

**Published:** 2023-11-30

**Authors:** Elana M. Meijer, Rachel Giles, Christian G. M. van Dijk, Ranganath Maringanti, Tamar B. Wissing, Ymke Appels, Ihsan Chrifi, Hanneke Crielaard, Marianne C. Verhaar, Anthal I.P.M. Smits, Caroline Cheng

**Affiliations:** † Department of Nephrology and Hypertension, Division of Internal Medicine and Dermatology, University Medical Center Utrecht, Utrecht 3508 GA, The Netherlands; ‡ Regenerative Medicine Center Utrecht, University Medical Center Utrecht, Utrecht 3508 GA, The Netherlands; § Experimental Cardiology, Department of Cardiology, Thorax Center Erasmus University Medical Center, Rotterdam 3000 CA, The Netherlands; ● Department of Biomedical Engineering, Eindhoven University of Technology; Eindhoven 5612 AE, The Netherlands; ⬡ Institute for Complex Molecular Systems (ICMS), Eindhoven University of Technology; Eindhoven 5612 AE, The Netherlands; □ Department of Biomedical Engineering, Erasmus Medical Center, Rotterdam 3000 CA, The Netherlands

**Keywords:** vascular smooth muscle cells, blood vessels, iPSCs, organoids, vasculature, GelMa, tissue engineering, hydrogels

## Abstract

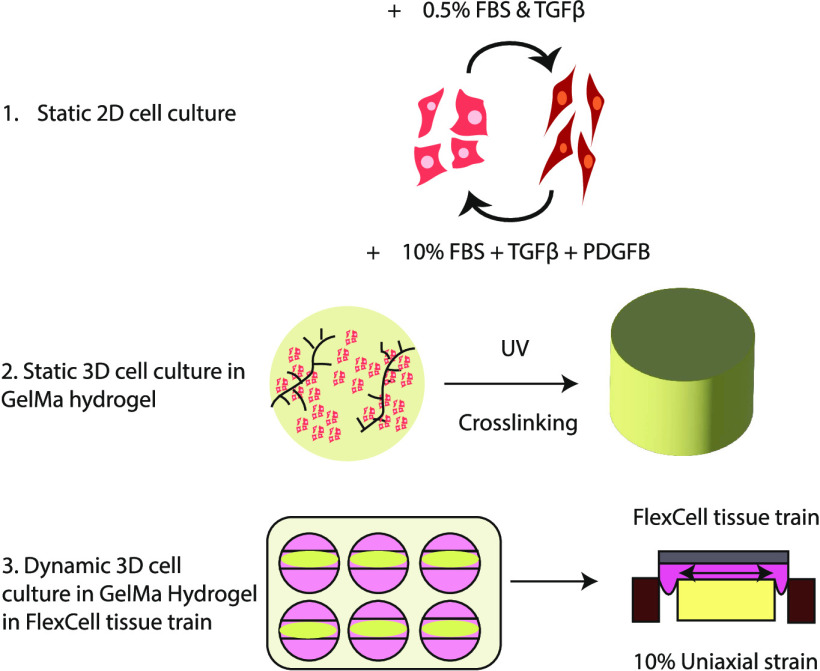

**Introduction:** Vascular smooth muscle cells (VSMCs) play a pivotal role in vascular homeostasis, with dysregulation leading to vascular complications. Human-induced pluripotent stem-cell (hiPSC)-derived VSMCs offer prospects for personalized disease modeling and regenerative strategies. Current research lacks comparative studies on the impact of three-dimensional (3D) substrate properties under cyclic strain on phenotypic adaptation in hiPSC-derived VSMCs. Here, we aim to investigate the impact of intrinsic substrate properties, such as the hydrogel’s elastic modulus and cross-linking density in a 3D static and dynamic environment, on the phenotypical adaptation of human mural cells derived from hiPSC-derived organoids (ODMCs), compared to aortic VSMCs. **Methods and results:** ODMCs were cultured in two-dimensional (2D) conditions with synthetic or contractile differentiation medium or in 3D Gelatin Methacryloyl (GelMa) substrates with varying degrees of functionalization and percentages to modulate Young’s modulus and cross-linking density. Cells in 3D substrates were exposed to cyclic, unidirectional strain. Phenotype characterization was conducted using specific markers through immunofluorescence and gene expression analysis. Under static 2D culture, ODMCs derived from hiPSCs exhibited a VSMC phenotype, expressing key mural markers, and demonstrated a level of phenotypic plasticity similar to primary human VSMCs. In static 3D culture, a substrate with a higher Young’s modulus and cross-linking density promoted a contractile phenotype in ODMCs and VSMCs. Dynamic stimulation in the 3D substrate promoted a switch toward a contractile phenotype in both cell types. **Conclusion:** Our study demonstrates phenotypic plasticity of human ODMCs in response to 2D biological and 3D mechanical stimuli that equals that of primary human VSMCs. These findings may contribute to the advancement of tailored approaches for vascular disease modeling and regenerative strategies.

## 1 Introduction

Vascular smooth muscle cells (VSMCs) provide structural vascular support and stability, regulate blood flow, contribute to immune responses, and participate in tissue repair mechanisms.^1−3^ VSMC dysfunction leads to vascular complications with a significant impact on morbidity and mortality, including (non)obstructive coronary artery disease (CAD), carotid artery and aortic aneurysms, and pulmonary arterial hypertension.^4−8^ Understanding the cellular processes that contribute to disease etiology is essential for developing dedicated *in vitro* disease models, targeted therapies, as well as regenerative strategies for the treatment of vascular diseases.

Following differentiation during vascular development, VSMCs retain a considerable degree of plasticity, manifesting a spectrum of phenotypic variations. This spectrum encompasses synthetic characteristics, including proliferation, extracellular matrix (ECM) synthesis, and tissue repair, as well as contractile properties involving force generation.^6,9,10^ During neovascularization, VSMCs exhibit a synthetic phenotype characterized by high rates of proliferation, cell migration, and deposition of ECM.^3,4^ In mature functional blood vessels, VSMCs typically assume a contractile phenotype to fulfill an essential role in vessel stabilization and vasomotion, displaying a quiescent state with an elongated, spindle-shaped morphology.^4^ Phenotypic switching between contractile to synthetic occurs during adulthood in response to, *e.g*., vessel injury and represents a critical step in the repair process.^5^

Although animal models and primary VSMC culture systems have provided valuable insights into vascular biology and disease, there are substantial disparities in the vascular physiology between rodents and humans. Additionally, limited availability of patient-derived VSMCs and the absence of well-characterized patient-specific three-dimensional (3D) tissue models that aim to closely mimic the dynamic *in vivo* environment currently impede advancements in research. Human-induced pluripotent stem-cell (iPSC)-derived VSMCs offer an alternative platform for studying human vascular biology.^11−17^ Generated from healthy donor- or patient-derived somatic cells, iPSC-derived tissue cells represent an abundant source for disease modeling, drug screening, and tissue engineering. Concerning VSMCs derived from iPSCs, several significant challenges remain to be addressed. One key issue is the phenotypic and developmental heterogeneity observed in many culture protocols, resulting in a mixed population of VSMCs with varying levels of synthetic and contractile phenotypes as well as a degree of “contamination” of the cell pool with non-VSMCs. Several strategies have been developed for the enrichment of lineage- or phenotype-specific VSMCs while excluding non-VSMCs.^18,19^ However, most published studies have still relied on differentiated VSMCs with an unclear embryonic origin, purity, or functional phenotype. This phenotypic heterogeneity, in particular, poses a significant challenge in their application for human disease modeling as well as regenerative medicine. The successful replication of physiological function relies on the presence of contractile VSMCs, whereas in vascular diseases as well as tissue (re)generation, synthetic VSMCs play critical roles. This duality becomes particularly problematic as iPSC-derived VSMCs need to effectively mimic either contractile or synthetic phenotypes to accurately represent various disease conditions *in vitro* or achieve the desired cellular phenotypes in different phases of vascular tissue engineering. An improved understanding of how environmental factors define (h)iPSC-derived VSMC phenotypes could provide leads to possible solutions.

Various biological growth factors are known to induce the phenotypic switch in VSMCs *in vitro*, including Platelet-Derived Growth Factor B (PDGFB), which can be used to induce synthetic characteristics in primary VSMCs,^18,20^ and transforming growth factor β (TGF-β), which is reported to reverse synthetic VSMCs into a contractile phenotype.^19^ In addition, the phenotype transformation of VSMCs is known to be modulated by mechanical cyclic strain, as shown in various *in vitro* studies, mimicking *in vivo* vascular dynamics. VSMC responses are variable, depending on the applied strain frequencies, elongation, and orientation (*e.g*., uni- or bidirectional, on a flat or circumferential surface).^21−23^ Phenotype determination in primary VSMCs may also be controlled by intrinsic matrix properties,^24,25^ where cell responses appear to significantly differ between 2D (cells grown on top of a matrix substrate) and 3D (grown in a matrix substrate) culture conditions.^26−28^

For (h)iPSC-derived VSMCs, previous studies have demonstrated that both biological growth factors and dynamic strain can initiate phenotypic adaptation, resembling the response of primary VSMCs.^14,22^ Nevertheless, the influence of intrinsic matrix substrate properties, such as the hydrogel’s elastic modulus, and cross-linking density (degree of functionalization, or DOF) on (h)iPSC-derived VSMCs, especially in 3D instead of 2D structures, where the 3D environment closely mimics the physiological conditions, has not yet been investigated. Moreover, the phenotypic adaptation of (h)iPSC-derived VSMCs in response to these factors in 3D environments under cyclic strain remains unexplored.

In this study, we evaluated the potential of human mural cells derived from hiPSCs obtained from vascular organoids (organoid-derived mural cells, or ODMCs) to adapt into VSMCs with a contractile or synthetic phenotype. In particular, their responses to various phenotype differentiation inducers, such as biological growth factors (TGF-β and PDGFB), were evaluated. Additionally, we assessed their phenotypic responses to differences in elastic modulus and cross-linking density of the matrix in a 3D environment, using Gelatin Methacryloyl (GelMa) with weight percentages and DOFs, respectively, in the presence and absence of cyclic unidirectional strain. By investigating the impact of these environmental components on the phenotypic switch in ODMCs, we can define the optimal conditions to grow and maintain a hiPSC-derived VSMC pool with the desired phenotype. The findings from this study thus provide valuable strategies for complex *in vitro* modeling of vascular diseases and have implications for regenerative approaches.

## 2 Methods

### 2.1 2D Growth Factor Experiments

#### 2.1.1 2D Cell Culture

ODMCs were differentiated and harvested from hiPSC-derived blood vessel organoids as described previously.^29^ Aortic VSMCs were purchased from Lonza. Both ODMCs and VSMCs were cultured on 1% gelatin-coated cell culture plates in SMGM-2 medium (Lonza) at 5% CO_2_. The medium was changed every other day. Cells were passaged for expansion or harvesting using Trypsin/EDTA (Gibco). The cells were used until passage 7.

#### 2.1.2 Growth Factor-Induced Phenotypic Switch

Cells were plated onto gelatin-coated 18 mm coverslips (staining) or gelatin-coated 6-well plates (PrestoBlue and gene expression analysis). They were serum-starved (0.5% Fetal Bovine Serum (FBS) in DMEM) for 24 h before the phenotypic switch. Control groups were cultured in DMEM with 10% FBS and 1% Pen/Strep (P/S). Synthetic groups were cultured in DMEM with 10% FBS, 1% P/S, 10 ng/mL PDGF, and 1 ng/mL TGFβ. Contractile groups were cultured in DMEM with 0.5% FBS, 1% P/S, and 1 ng/mL TGFβ. All were kept at 37 °C with 5% CO_2_.

#### 2.1.3 PrestoBlue Viability Assay

Cells (*n* = 6 different vials per cell type) were seeded on a gelatin-coated six-well plate with a cell density of 50 000 cells per well. Cell viability was measured 24, 72, and 144 h after growth factor treatment using PrestoBlue Cell Viability Reagent (Thermo Scientific) according to the manufacturer’s protocol.

#### 2.1.4 FACS Analysis of ODMCs

ODMCs were cultured and harvested using Trypsin/EDTA as described above. Cells were distributed in a 96-well plate (25 000 cells per well) and subsequently stained with anti-CD31 and anti-CD140b antibodies (Table S1), together with Sytox blue (Invitrogen) to exclude dead cells. The CytoFLEX flow cytometer (Beckman Coulter) was used for cell analysis, and data analysis was performed using FlowJo software (Version 10.2).

### 2.2 3D Static GelMa Experiments

#### 2.2.1 GelMa Hydrogel Preparation

Two GelMa stocks with different degrees of Functionalization (DOF) were prepared. For both, 10 g of type A gelatin from porcine skin (Sigma-Aldrich) was dissolved in 100 mL of phosphate-buffered saline (PBS) at 60 °C to obtain a 10% gelatin solution. The DOF is defined by the percentage of modified lysin residues as described and validated previously.^30,31^ After 3 h, 400 mL of PBS was added and the solution was dialyzed against distilled water to remove salts and methacrylic acid for 7 consecutive days. Finally, the solution was lyophilized and stored at −80 °C until further use.

To prepare hydrogels, GelMa dissolved in PBS was subjected to radical cross-linking in the presence of a photoinitiator. For this, a 0.1% 2-hydroxy-2-methylpropiophenone photoinitiator (PI) (Irgacure, Sigma-Aldrich) was prepared using PBS. Lyophilized GelMa (5 or 10% w/v) was mixed with 0.1% PBS–PI and incubated for 15 min at 80 °C to dissolve. This protocol is executed according to a previously described protocol by Gartner et al.^32^

#### 2.2.2 GelMa Swelling Assay

30 μL of prepolymer solution with both 80 DOF and 50 DOF (5 and 10%) was pipetted onto a 10 cm Petri dish between two spacers with a height of 0.45 mm and was covered with a sterile glass slide. The prepolymer solution was placed under a 450 mW ultraviolet (UV)-light (OmniCure Series 2000, Excelitas) for 50 s. The hydrogel was removed from the glass slide and washed with PBS. Empty hydrogels were incubated in PBS at 37 °C for 24 h before mechanical testing and hydrogel swelling analysis.

Swollen GelMa hydrogels (3 per experiment for 5 different experiments) were weighed (ww) and subsequently dried by lyophilization. After that, dried weight (wd) of GelMa hydrogels was obtained and the mass-swelling ratio (*q*) was calculated as *q* = ww/wd.

#### 2.2.3 Dynamic Mechanical Analysis (DMA)

The Q800 Dynamic Mechanical Analyzer (DMA) (TA Instruments, Inc.) was used to test the hydrogel mechanical properties through a controlled force. The hydrogels were placed between the parallel-plate compression. A ramp force was applied at 0.010 N/min to 0.500 N with a preload force of 0.0010 N for 10 min. The Young’s modulus was calculated by the slope of the most linear part (8 data points) of the stress–strain curve.

#### 2.2.4 Cell-Laden 3D Static GelMa Hydrogels

The ODMCs/VSMCs were added to the GelMa-PI solution to achieve a cell density of 75.000 cells per 30 μL. The cell containing GelMa solution (30 μL) was pipetted on a 10 cm Petri dish between two spacers covered with a sterile microscope slide and placed under a 450 mW UV light (OmniCure Series 2000, Excelitas) for 50 s. The hydrogels were subjected to serum starvation (0.5% DMEM) for 24 h, after which 10% DMEM was added. *N* = 6 per condition.

#### 2.2.5 Live/Dead Cell Viability Assay

A live/dead assay on the cell-laden GelMa constructs (*n =* 6 per condition) was performed using a LIVE/DEAD Cell Imaging kit (Invitrogen, Waltham, Massachusetts) 144 h after GelMa synthesis. The fluorescent dyes were diluted according to the manufacturer’s protocol, in DMEM supplemented with 10% FBS and 1% P/S and added to the hydrogels to incubate for 10 min at room temperature in the dark. The constructs were analyzed with fluorescence imaging on 470 and 550 nm wavelengths for the green and red signals, respectively.

### 2.3 3D Dynamic Experiments

#### 2.3.1 Cell-Laden 3D GelMa Hydrogels Exposed to Dynamic Loading

Two 5 mm × 20 mm Velcro strips were glued in parallel, 5 mm apart, to the bottom of each well of 6-well Bioflex culture plates (untreated, Flexcell Int) using medical adhesive silicone (Silastic MDX4–4210, Dow Corning, Midland, MI). Each pair of Velcro strips served as a mold to attach a hydrogel to the Flexcell membrane. Cell-GelMa suspensions (750 000 cells per 300 μL each) were pipetted within these molds and placed under a 450 mW UV light for 100 s. The hydrogels were covered with SMGM-2 medium and cultured for 2 days before dynamic loading was applied. On day 3, cell-laden GelMa hydrogels were placed on the Flexcell FX-5000T (Flexcell Int, McKeesport, PA) and exposed to 2 days of 10% strain (0.5 Hz). *N* = 3 per condition. A schematic representation of the strain experiments is displayed in Figure S2A.

#### 2.3.2 Strain Validation

To validate the intra- and interexperimental variations in dynamic loading, 3 × 3 dotted patterns were created on the membranes of a 6-well Bioflex culture plate (untreated, Flexcell Int). Videos were captured on days 3 (the first day of straining) and day 5. Subsequently, the maximum strain in the *y* direction (ε_*yy*,max_) was calculated (Figure S3) by tracking the displacements of the previously applied dotted patterns over time using the open-source software Tracker (https://physlets.org/tracker). A schematic representation of the strain validation is displayed in Figure S2D.

To validate whether hydrogel intrinsic properties affected the strain of cell-laden 3D GelMa hydrogels, 50–5 and 80–10 hydrogels (*n* = 6/group) without cells were created, covered with graphite particles, and imaged while being exposed to the 10% strain protocol. To assess whether cell remodeling activities would affect the strain pattern in the hydrogels, strain patterns of cell-laden 3D GelMa hydrogels were also assessed on day 5 in a similar fashion.

The captured videos were converted to images at 30 Hz in MATLAB (Mathworks, Massachusetts). Subsequently, the maximum strain in the *y* direction (ε_*yy*,max_) per loading regime group was calculated (Figure S3) using the open-source 2D DIC software Ncorr (v1.2, www.ncorr.com).

### 2.4 Analysis and Immunohistochemistry

#### 2.4.1 Quantitative Polymerase Chain Reaction Analysis

Total RNA was isolated from cultures (ODMCs and VSMCs) using an RNA isolation kit (Bioline) according to the manufacturer’s protocol. Cells from 3D GelMa constructs were extracted by using the QIAshredder columns according to the manufacturer’s protocol. The supernatant was subsequently used for RNA extraction with the RNA isolation kit from Bioline as described above. The purity and concentrations of RNA were quantified using spectrophotometry (DS-11; DeNovix) and absorbance measurements at 260/280 nm. cDNA synthesis was performed according to the protocol of the Bioline cDNA synthesis kit. Gene expression was determined using FastStart SYBR-green (Roche) following the quantitative polymerase chain reaction (qPCR) program: 8,5′ 95 °C, 38 cycles (15 in. 95 °C; 45 in. 60 °C) 1′ 95 °C, 1′ 65 °C, 62 cycles (10 in. 65 + 0.5 °C) in the SYBR-Green-Cycler IQ5 detection protocol (Biorad CFX384), performed in 384-well plates (Merck). The primer sequences used are listed in Table S2. All results were normalized for housekeeping genes ROPL and RPLP0, resulting in relative mRNA expression. In the dynamic experiments, results were compared to the static controls and represented as the fold change (ΔΔ*C*_t_).

#### 2.4.2 2D Immunohistochemistry

Phenotypic switch was induced in cells cultured on 18 mm coverslips as described in Section 2.1.2. Cells were fixated after 24 and 72 h using 4% PFA for 20 min. The cells were blocked using a 2% PBS/bovine serum albumin (BSA) solution for 30 min. The cells were stained with anti-Calponin overnight at 4 °C (Table S3). Thereafter, the staining solution was removed, and the coverslips were washed 3 times with PBS. Secondary antibody incubation together with phalloidin was performed for 1 h at RT (Table S3). The coverslips were washed with PBS and counterstained with DAPI for 5 min. Coverslips were mounted on microscope glass slides by using Mowiol 4–88. Samples were stored at 4 °C prior to imaging.

#### 2.4.3 3D Immunohistochemistry

Cell-laden GelMa constructs were fixated using 4% PFA for 1 h at RT. Constructs were blocked and permeabilized using 3% FBS, 1% BSA, 0.5% Triton X-100, and 0.5% Tween in PBS for 2 h at RT. GelMa constructs were stained with anti-Smooth Muscle Actin α and anti-Calponin (Table S3) for 2 h at RT. The cells were washed 3 times with PBS-/Tween, stained with secondary antibodies (Table S3), and incubated for 2 h at RT. DAPI was used as a counterstain, and GelMa constructs were mounted using Mowiol 4–88. Samples were stored at 4 °C prior to imaging.

#### 2.4.4 Imaging and Analysis

Imaging was performed using the Leica Confocal SP8× (2D cultures; 63× magnifications) and the Leica Thunder microscope (10, 20, and 40× magnifications for 3D GelMa constructs). Images were analyzed using ImageJ software (Version 1.47). 3D images were composed in LASX (Version 3.5.7.23225).

#### 2.4.5 Statistical Analysis

The statistical analyses were performed using GraphPad Prism (Version 8.3). Values are shown as individual data points with mean ± SEM. Prior to statistical testing, outliers were removed from the results when detected using Grubbs’ test (α = 0.05). The paired, two-sided *t* test and the ordinary one-way ANOVA test with the Tukey post hoc test were used when appropriate. Experiments were performed at least in triplicate. The detailed sample size for each result is listed in the legend of the figures. A *p*-value of *p* ≤ 0.05 was accepted as statistically significant. Significance is further described in the figure legends and the 3Section 3.

## 3 Results

### 3.1 ODMCs are Capable of Growth Factor-Induced Phenotype Switching Similar to Primary Human Aorta-Derived VSMCs

**Figure 1:**
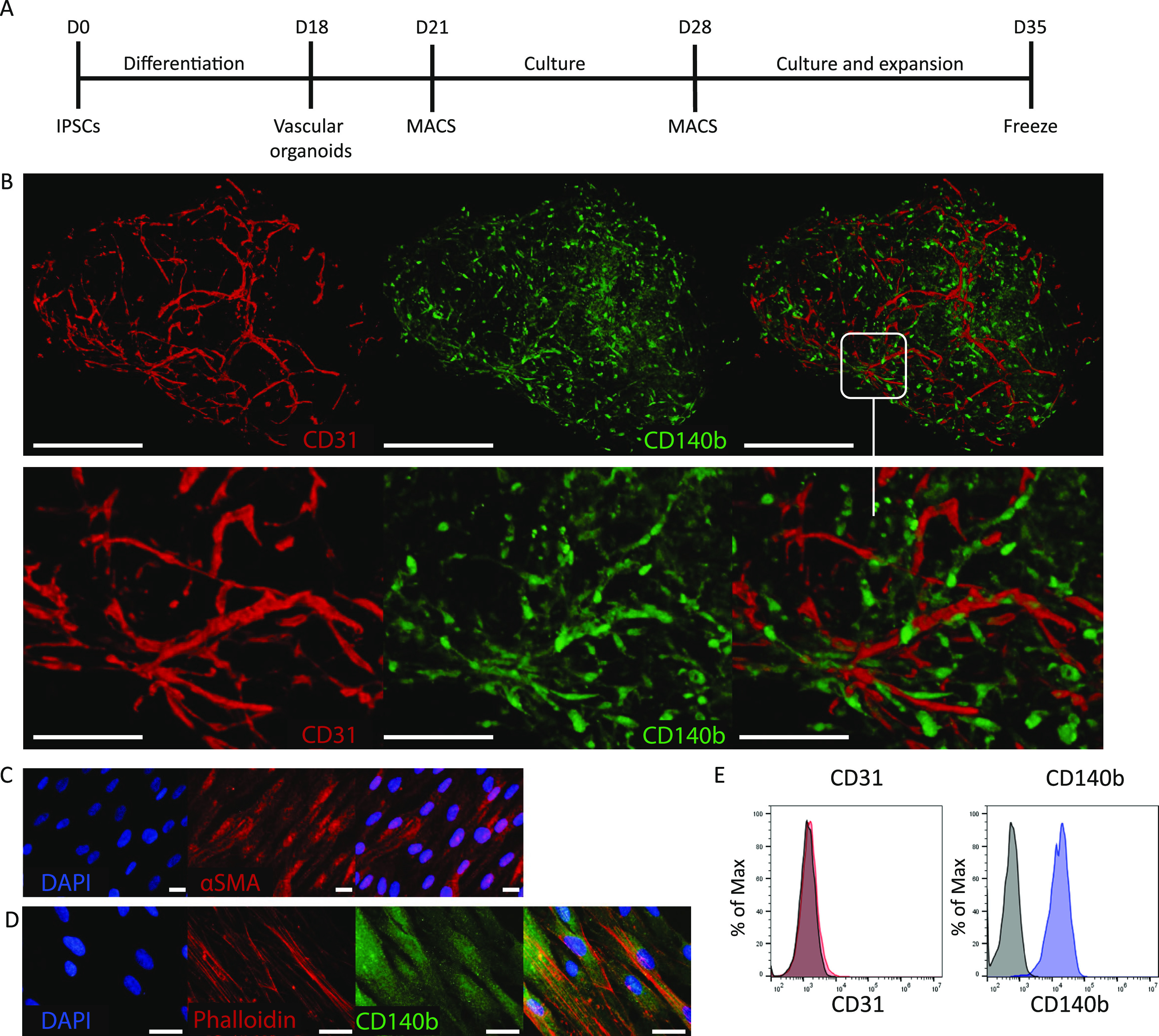
Differentiation of organoid-derived mural cells (ODMCs). (A**)** Schematic overview of the experimental timeline. (B) Whole mount staining analysis of a vascular organoid stained for both CD31 (red) and PDGFrβ (CD140b; green). Scale bar depicts 500 μm (complete organoid) and 100 μm (zoomed-in structure). (C) Immunofluorescent staining of ODMCs with anti-Smooth Muscle Actin-α (ACTA2; red) counterstained with DAPI (blue). Scale bar depicts 20 μm. (D) Immunofluorescent staining of ODMCs with anti-PDGFrβ (green) counterstained with phalloidin (red) and DAPI (blue). Scale bar depicts 20 μm. (E) FACS analysis of sorted ODMCs, stained for CD31 (red histogram) and CD140b (blue histogram), and compared to isotype control (gray).

ODMCs were harvested from human iPSC-derived blood vessel organoids following a previously described protocol summarized in Figure 1A. Blood vessel organoids were cultured following the adapted Wimmer lab protocol^29,33^ and contain both endothelial (CD31+) and mural (CD140b+) cells in a vasculature-like organization (Figure 1B). Extracted ODMCs brought into a single culture express both αSMA and CD140b (Figure 1C,E) and were devoid of endothelial cell contamination, as confirmed by FACS analysis (Figure 1E).

**Figure 2:**
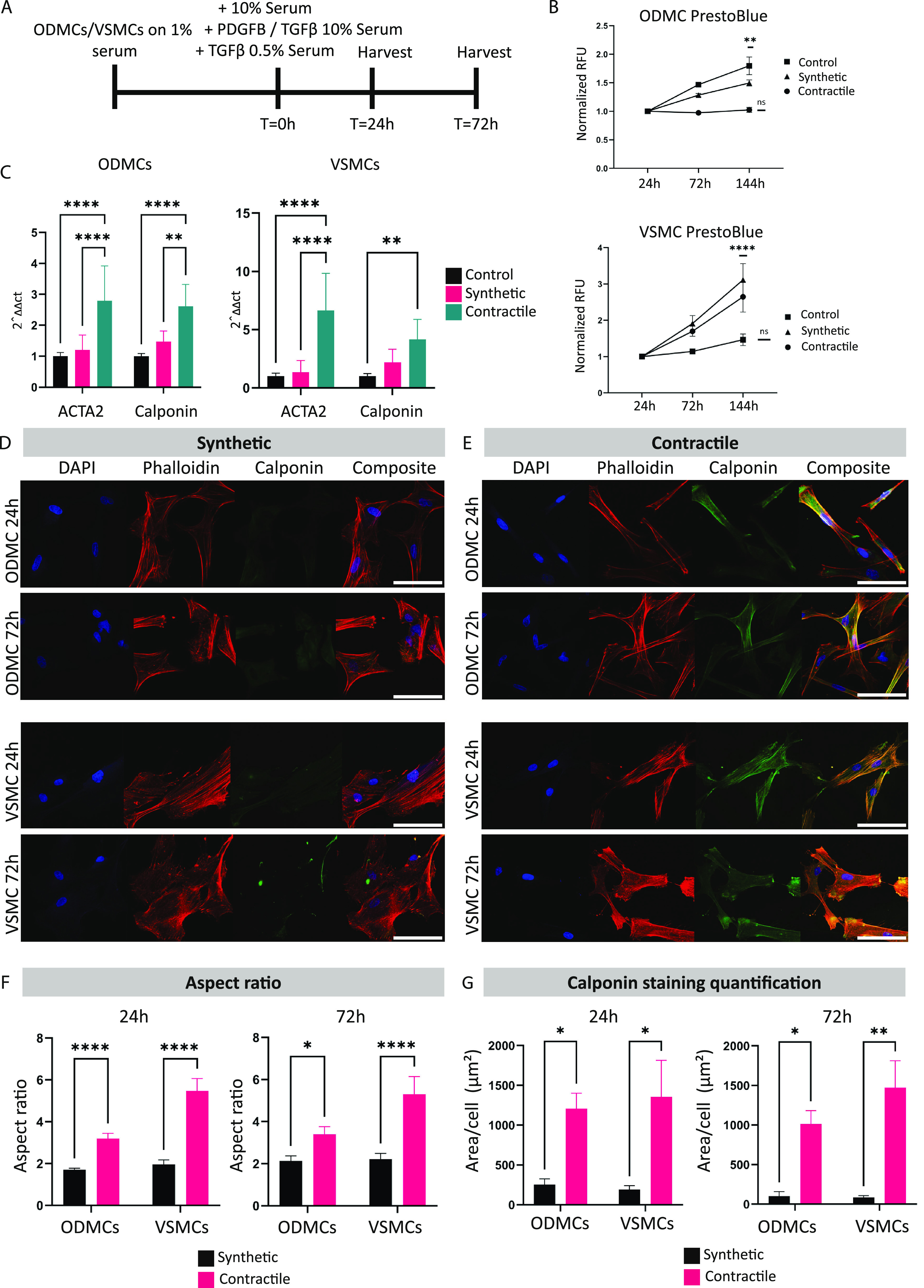
Growth factor-induced phenotypic switch in 2D. (A) Schematic overview of the experimental timeline. (B) PrestoBlue viability assay. Data represented as mean ± SEM, *n* = 6, one-way ANOVA with Tukey post hoc test. **p < 0.01 for ODMCs compared to 24 h and ****p < 0.0001 for VSMCs compared to 24 h. Data is normalized to the 24 h time point. (C) Gene expression analysis of the ODMCs and VSMCs 24 h after treatment normalized to the control conditions. Data represented as mean ± SEM, *n* = 6 for both cell types. One-way ANOVA with Tukey post hoc test, **p < 0.01, ****p < 0.0001. (D) 2D Immunofluorescent staining of synthetic ODMCs and VSMCs after 24 and 72 h, stained for contractile marker calponin (green), counterstained for phalloidin (red) and DAPI (blue). Scale bar depicts 20 μm. (E) 2D Immunofluorescent staining of contractile ODMCs and VSMCs after 24 and 72 h, stained for contractile marker calponin (green), counterstained for phalloidin (red) and DAPI (blue). Scale bar depicts 20 μm. (F) Aspect ratio of contractile and synthetic VSMC and ODMC populations after 24 and 72 h. Aspect ratio is calculated as major axis divided by the minor axis. *N* = 4 samples, 8 cells per sample. One-way ANOVA with Tukey post hoc test, *p < 0.05, ****p < 0.0001. (G) Calponin protein expression levels were determined by immunofluorescent quantification. Expression levels were calculated by the signal area divided by the number of nuclei. *N* = 6, one-way ANOVA with Tukey post hoc test, *p < 0.05, **p < 0.01.

Throughout the study, human aortic VSMCs were used as a control. The phenotypic switch toward a contractile phenotype in 2D culture was induced in VSMCs and ODMCs by a combination of low serum and TGFβ for 48 h (*T* = 72 h in culture, see schematics in Figure 2A). Full DMEM (with 10% serum) supplemented with both PDGFB and TGFβ was used for the synthetic phenotype, and the control groups were maintained on full DMEM (10% serum). Cell viability, as measured by PrestoBlue, remained unchanged in the contractile population, whereas in the control and synthetic groups, this number increased significantly over time for both VSMCs and ODMCs (Figure 2B). In addition, gene expression analysis of contractile markers ACTA2 and Calponin shows significant upregulation of both genes after inducing the phenotypic switch toward a contractile population, both in ODMCs and VSMCs (Figure 2C). Immunofluorescent staining of synthetic and contractile populations (Figure 2D,E) shows similar changes in the morphology and expression of Calponin protein levels in ODMCs and VSMCs in response to phenotype induction. The synthetic cells displayed a more rhomboid shape, while the contractile cells demonstrated cell elongation, as indicated by quantification of the aspect ratio (major axis/minor axis; Figure 2F). Cell expression of the Calponin protein was quantified by assessment of the Calponin+ area per cell (Figure 2G). The contractile populations of ODMCs and VSMCs show comparable high levels of Calponin+ cells, compared to their respective synthetic populations. This data indicates that ODMCs are capable of adapting to a synthetic or contractile phenotype induced by different growth factor regimes, similar to primary VSMCs.

### 3.2 ODMC and VSMC Static Culture in 3D in Different GelMa Hydrogel Conditions with Specific Intrinsic Matrix Characteristics Has Limited Impact on Cell Survival

**Figure 3:**
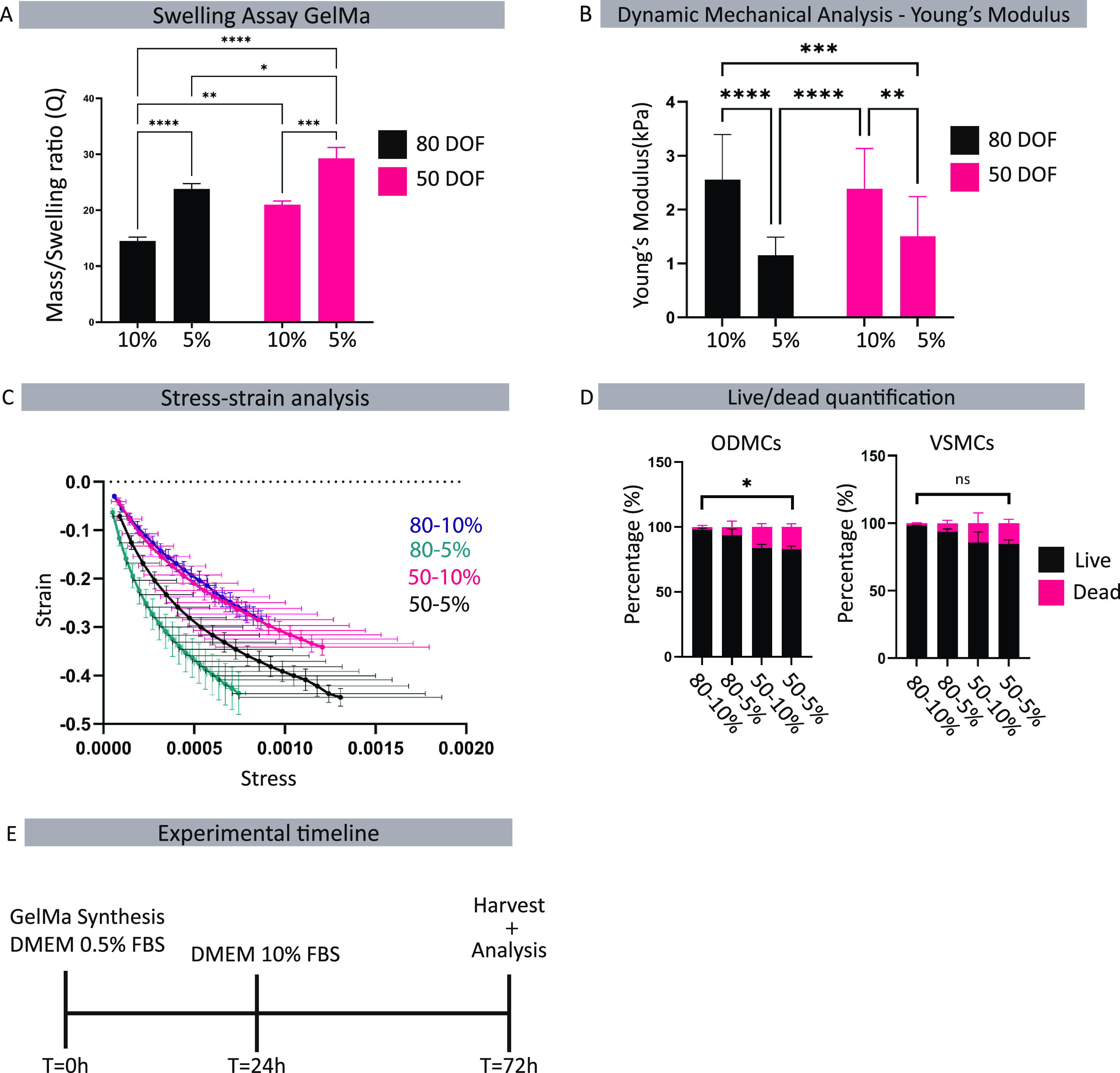
ODMCs and VSMCs in 3D GelMa hydrogels. (A) Swelling assay; displayed as mass/swelling ratio (Q) for both 5 and 10% gels of 80 DOF and 50 DOF. Data represented as mean ± SEM, *n* = 5, one-way ANOVA with Tukey post hoc test, *p < 0.05, **p < 0.01, ***p < 0.001, ****p < 0.0001. (B) Young’s modulus of the GelMa hydrogels, displayed in kPA. Data represented as mean ± SEM, *n* = 5, one-way ANOVA with Tukey post hoc test, **p < 0.01, ***p < 0.001. **C**. Stress–strain curves of the GelMa hydrogels. *N* = 20 gels per condition. (D) Live–dead assay of the vascular cells in GelMa hydrogels. Data represented as percentage live or dead cells and displayed as mean ± SEM, *n* = 5, one-way ANOVA with Tukey post hoc test, *p < 0.05. (E) Schematic overview of the experimental timeline.

Cells were seeded and statically cultured in 3D in GelMa hydrogels for a total of 72 h before harvesting (Figure 3E). Bare GelMa hydrogel characteristics were first analyzed, starting with the water absorption capacity, using a swelling assay (Figure 3A). This assay tests the DOF, with higher DOF hydrogels accommodating a higher degree of cross-linking, resulting in a lower mass/swelling ratio (*q*). The *q* was higher for the 5% compared to the 10% hydrogels for both DOFs (*p* < 0.001 for 50 DOF, *p* < 0.0001 for 80 DOF). All of the 80 DOF hydrogels show significantly higher ratios versus 50 DOF when compared to their respective percentage counterparts.

The hydrogel mechanical characteristics were assessed by dynamic mechanical analysis (DMA), generating stress–strain curves from which Young’s modulus was calculated and displayed as kPA (Figure 3B,C). The Young’s modulus reflects the material’s viscoelastic response, with higher values representing higher material resistance to deformation when subjected to mechanical forces. The 10% hydrogels demonstrate a significantly higher Young’s modulus compared to the 5% hydrogels for all DOFs (*p* < 0.0001 for 80 DOF, *p* < 0.01 for 50 DOF).

Calculation of the Live/dead cell ratio of ODMCs and VSMCs in the different hydrogels after a maximum of 144 h of static culture shows no effect of hydrogel conditions in ODMCs and only a significant decline in the 5% 50 DOF versus the 10% 80 DOF condition in VSMCs (Figure 3D). Combined, this data validates the differences in intrinsic matrix properties between hydrogel conditions. The higher water absorption capacity in the 50 DOF compared to the 80 DOF indicates a higher cross-linking density in the 80 DOF. Additionally, there was a higher Young’s modulus in the 10% versus the 5% compositions. These conditions have no significant impact on the cell survival of ODMCs in static culture.

### 3.3 3D GelMa Hydrogel with Higher DOF Combined with Lower Young’s Modulus Promotes a Contractile Phenotype in ODMCs and VSMCs under Static Conditions

**Figure 4:**
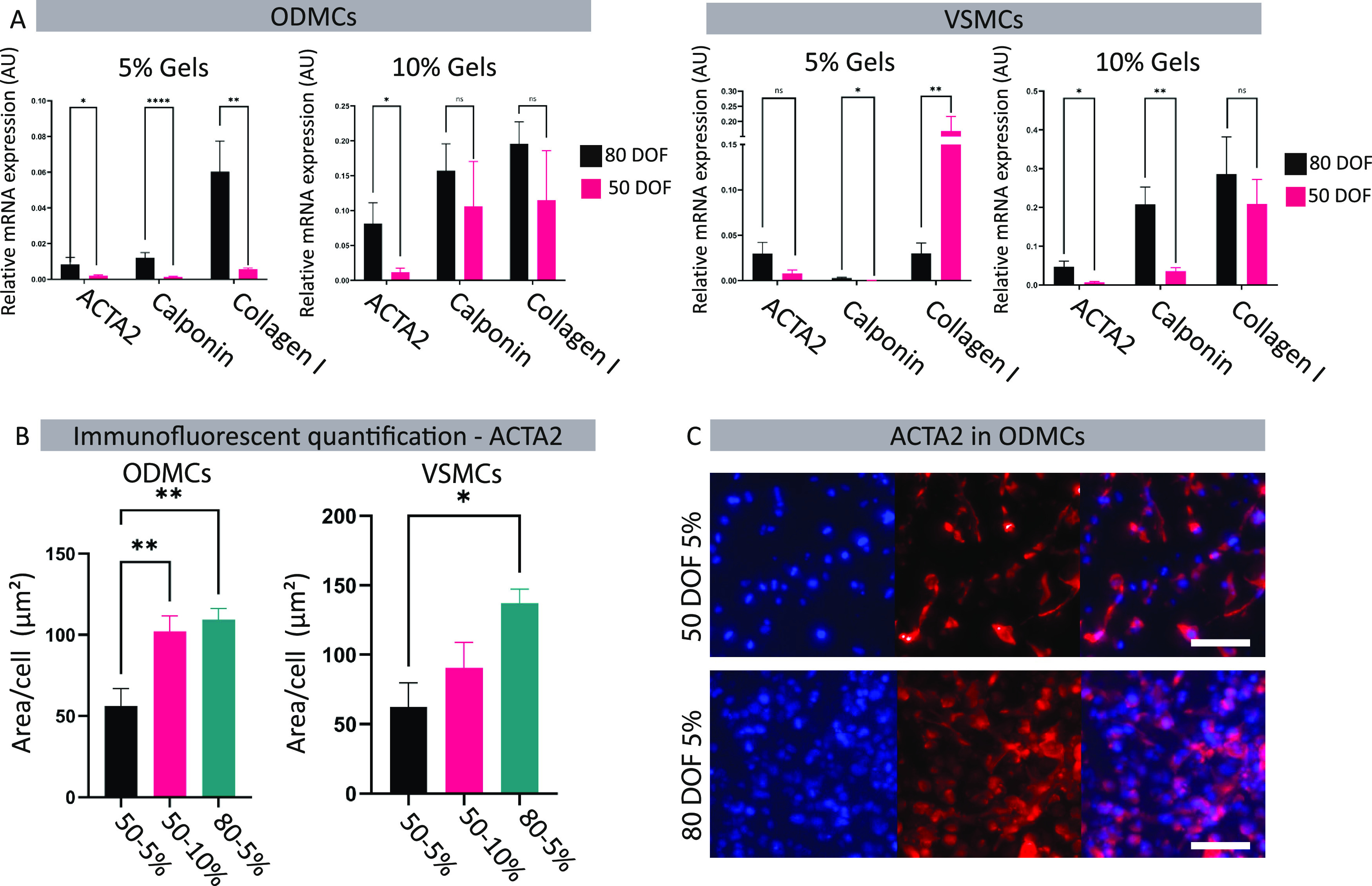
Effect of GelMa properties on smooth muscle cell phenotype in static conditions. (A) Gene expression analysis of the ODMCs and VSMCs 48 h after seeding. Data represented as mean ± SEM, *n* = 6 for ODMCs, *n* = 5 for VSMCs. One-way ANOVA with Tukey post hoc test, *p < 0.05, **p < 0.01, ***p < 0.001. (B) ACTA2 protein levels were based on immunofluorescent quantification. Expression levels were calculated by the signal area divided by the number of nuclei. *N* = 5 gels, 3 locations per gel, one-way ANOVA with Tukey post hoc test, *p < 0.05, **p < 0.01, ****p < 0.0001. (C) Immunofluorescent whole mount staining of ACTA2 (red). Stained GelMa gels were obtained 48 h after seeding. DAPI (blue) was used as a counterstain. Scale bar depicts 50 μm.

After 72 h of static 3D culture in the GelMa hydrogels, cells were harvested for analysis. Expression of contractile markers ACTA2, Calponin, and Collagen I was assessed using qPCR to evaluate the impact on cell phenotype. Relative mRNA expression levels are shown in Figure 4A. In ODMCs, in 5% hydrogels, 80 DOF had significantly increased expression of contractile markers compared to 50 DOF (*p* < 0.05 for ACTA, *p* < 0.0001 for Calponin, and *p* < 0.01 for Collagen I). In 10% hydrogels, a similar trend was observed, where ACTA2 expression was significantly increased in 80 DOF versus 50 DOF (*p* < 0.05) (Figure 4A). This indicates the GelMa hydrogels with a similar Young’s modulus, but higher cross-linking densities (DOF) promoted more adaptation to a contractile phenotype under static culture in ODMCs. For VSMCs, a similar trend was observed with increased expression of Calponin, as well as a decrease in Collagen I, in the 80 DOF compared to 50 DOF in the 5% hydrogels (*p* < 0.05 for Calponin, *p* < 0.01 for Collagen I). In the 10% hydrogels, both ACTA2 and Calponin were upregulated in the 80 DOF versus 50 DOF (*p* < 0.05 for ACTA, *p* < 0.01 for Calponin).

When the weight percentages of the same DOFs were compared, a significant (*p* < 0.05) increased expression was observed in the ODMCs of both Calponin and Collagen I in the 10% hydrogels compared to the 5% hydrogels of the same DOFs (Figure S1A). For VSMCs, there were no significant differences in the expression of contractile genes under the 50 DOF conditions. In the 80 DOF, the 10% hydrogels caused significant (*p* < 0.05) upregulation of Calponin and Collagen I. These findings indicate that 3D culture in GelMa hydrogels with the same DOF and a higher Young’s modulus promotes a contractile phenotype in ODMCs and VSMCs.

The addition of growth factors under the different static 3D GelMa conditions did not significantly alter gene expression of contractile markers of ODMCs or VSMCs (Figure S1B). Protein levels of ACTA2, quantified by the assessment of the ACTA2+ area per cell (Figure 4B), showed a significant increase in 80 versus 50 DOF in 5% hydrogels for ODMCs and an increase in 80 versus 50 DOF, similarly, in 5% hydrogels for VSMCs. Examples of the ACTA2 staining of the 50 and 80 DOF in 5% hydrogel for ODMCs are shown in Figure 4C. Examples of ACTA2 staining for all conditions for both ODMCs and VSMCs are displayed in Figure S1C. For 80 DOF in 10% hydrogel, both ODMCs and VSMCs exhibited a rounded cell morphology with limited elongation, suggesting minimal interaction with the hydrogel.

### 3.4 Uniaxial Strain Induces a Switch toward a Contractile Phenotype in 3D GelMa Cultured ODMCs and VSMCs

**Figure 5:**
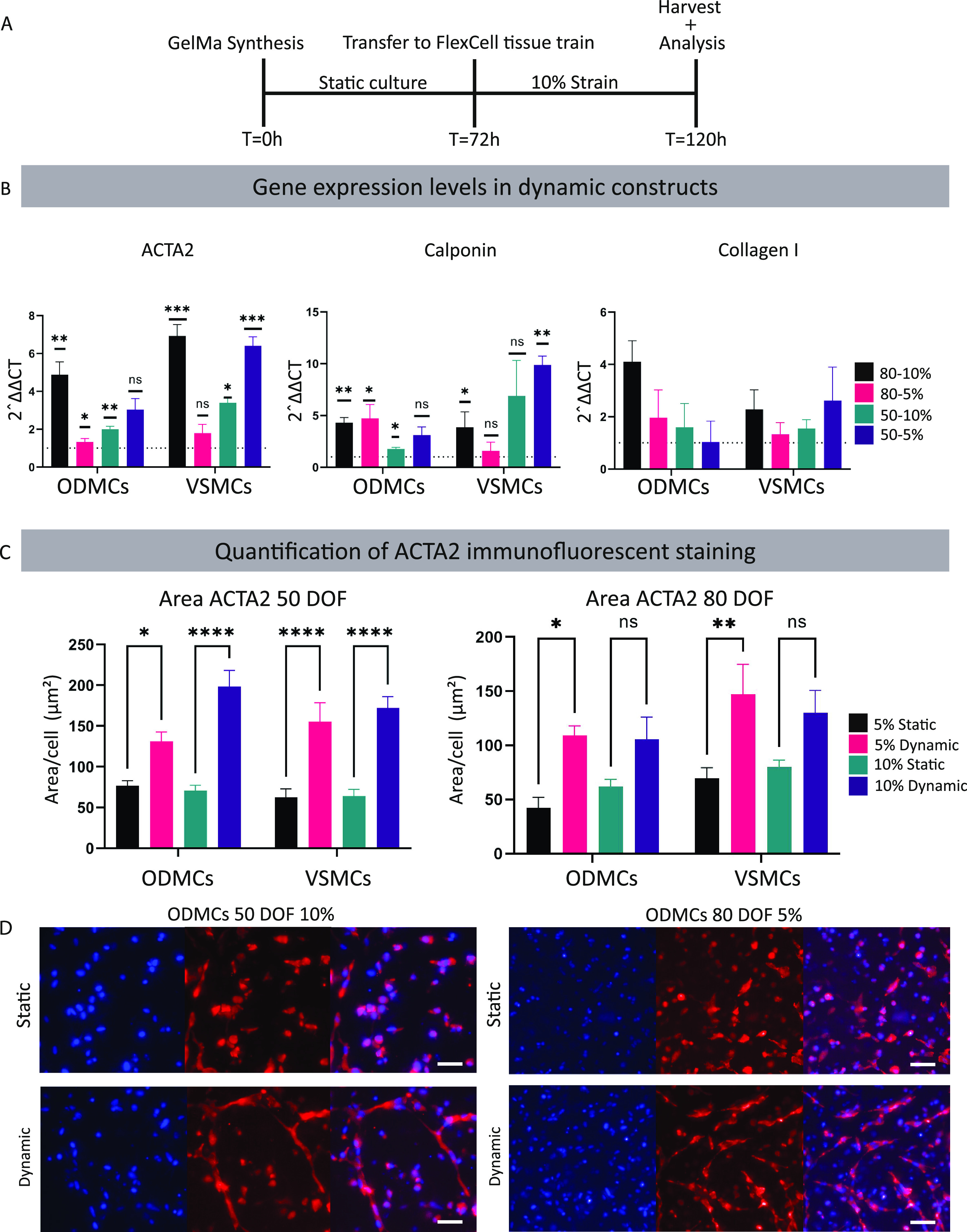
Effect of cyclic strain on the smooth muscle cell phenotype in 3D GelMa hydrogels. (A) Schematic overview of the experimental timeline. (B) Gene expression analysis of the ODMCs and VSMCs after 48 h of 10% strain. Results are compared to the static controls (dotted line). Data represented as mean ± SEM, *n* = 3 for both conditions. One-way ANOVA with Tukey post hoc test, *p < 0.05, **p < 0.01, ***p < 0.001. (C) ACTA2 protein levels were based on immunofluorescent quantification. Expression levels were calculated by the signal area divided by the number of nuclei. *N* = 3 gels, 3 locations per gel, one-way ANOVA with Tukey post hoc test, *p < 0.05, **p < 0.01, ****p < 0.0001. (D) Immunofluorescent whole mount staining of the static and dynamic (48 h of 10% strain) GelMa gels. Cells were stained for ACTA2 (red), and DAPI (blue) was used as a counterstain. Scale bar depicts 50 μm.

Using the Flexcell© Tissue train system, 10% uniaxial strain was applied for 48 h on (72 h old) seeded hydrogels, as displayed in the timeline in Figure 5A. The effect of hydrogel characteristics on strain patterns was assessed by comparing strain levels between the strongest (80 DOF in 10%) and weakest (50 DOF in 5%) hydrogels. No significant differences in strain patterns were detected (Figure S2B). Strain analysis also reveals no significant differences in strain levels between day 0 and day 2 time points or between the different experiments (Figure S2C). Gene expression levels of contractile markers under dynamic conditions were compared to the static controls, as displayed in Figure 5B. After 48 h of strain, the expression of contractile markers increased in GelMa hydrogels for multiple conditions. In ODMCs, 80 DOF in 5 and 10%, and 50 DOF in 10% GelMa hydrogels show significantly increased expression of ACTA2 (*p* < 0.01 for 80 DOF in 10%, *p* < 0.05 for 80 DOF in 5% and *p* < 0.01 for 50 DOF in 10% hydrogel) and Calponin (*p* < 0.01 for 80 DOF in 10%, *p* < 0.05 for 80 DOF in 5% and *p* < 0.05 for 50 DOF in 10% hydrogel) after exposure to strain. For VSMCs, all conditions except the 80 DOF in 5% hydrogel showed significantly increased expression of ACTA2 (*p* < 0.001 for 80 DOF in 10%, *p* < 0.05 for 50 DOF in 10%, and *p* < 0.001 for 50 DOF for 5% hydrogel). For Calponin, only the cells in the 50 DOF in 5% (*p* < 0.05) and 80 DOF in 10% hydrogels (*p* < 0.01) showed significant upregulation. For Collagen I, the exposure to strain did not significantly affect gene expression levels in both cell types. ACTA2 protein levels were assessed by quantification of the ACTA2+ area per cell (Figure 5C). Strain significantly increased ACTA2+ levels in the 50 DOF hydrogels for both cell types (*p* < 0.05 for 5% hydrogels in ODMCs, *p* < 0.001 for 5% hydrogels in VSMCs and for 10% hydrogels in VSMCs and ODMCs).

In the 80 DOF hydrogels, there was only a significant increase in the 5% hydrogels in both cell types (*p* < 0.05 for ODMCs, *p* < 0.01 for VSMCs). Examples of the ACTA2 staining of both 50 DOF in 10% and 80 DOF in 5% hydrogels are shown in Figure 5D. Increased elongation of both ODMCs and VSMCs after exposure to strain is clearly visible, indicating morphological adaptation to strain. ACTA2 staining for all conditions for both cell types is displayed in Figure S3A,B.

## 4 Discussion

Within the vascular research field, there is a notable gap in comparative studies regarding the impact of intrinsic matrix substrate properties, such as elastic modulus and degree of cross-linking, on the cell behavior of (h)iPSC-derived VSMCs, particularly when cultured in 3D structures. Additionally, the phenotypic adaptation of (h)iPSC-derived VSMCs in response to these factors in 3D environments under cyclic strain is largely unexplored. Here, we demonstrated the ability of hiPSC ODMCs to undergo phenotype switching under different culture and 3D GelMa hydrogel conditions, similar to primary VSMCs, illustrating their suitability for use in modeling of complex diseases and potential for therapeutic interventions. The main findings are (1) ODMCs derived from hiPSCs exhibited a VSMC phenotype, expressing key mural markers such as α-smooth muscle actin (αSMA) and CD140b. (2) ODMCs demonstrated phenotypic plasticity in response to specific culture conditions and can adopt a contractile phenotype similar to primary human VSMCs. (3) The mechanical properties in a 3D hydrogel substrate, including elastic modulus and degree of cross-linking had profound impact on both ODMCs and VSMCs under static culture, where hydrogels with a higher Young’s modulus and a higher cross-linking density induced a contractile phenotype. (4) Dynamic stimulation in a 3D substrate using uniaxial strain further promotes a switch toward a contractile phenotype in both ODMCs and VSMCs. Our research enhances our knowledge of human iPSC-derived VSMCs, particularly ODMCs, by elucidating the influence of culture medium composition, intrinsic matrix properties, and dynamic stimuli on phenotypic changes. These findings have practical implications for tissue engineering and regenerative medicine, especially in vascular disease treatment and modeling.

### 4.1 ODMCs Are Capable of Growth Factor-Induced Phenotype Switching Similar to Primary Human Aorta-Derived VSMCs

ODMCs derived from vascular organoids^33^ typically exhibit pericyte-like coverage of microcapillaries. However, when isolated and purified through CD140b sorting and cultured in VSMC medium, they undergo a morphological transition toward VSMC-like cells. When seeded on solution electrospun vascular scaffolds, these ODMCs can form tissue structures resembling the tunica media.^29^ Notably, they form a distinct multicellular layer separate from the endothelium and contribute to the stability of these vascular grafts under flow conditions. The phenotypic characteristics of these ODMCs have not been comprehensively evaluated. Here, we demonstrate, for the first time, the phenotypic plasticity of ODMCs derived from vascular organoids.

In healthy adult vasculature, VSMCs exhibit a contractile phenotype with limited proliferation and low synthetic activity. Following vascular injury, VSMCs undergo phenotypic changes, increasing migratory, synthetic, and proliferative capacities, contributing to vascular repair but also to diseases such as atherosclerosis, cancer, and hypertension.^9,34,35^ Notably, PDGFB induces the synthetic VSMC phenotype by downregulating contractile gene expression and promoting proliferation and migration.^1,36^ In contrast, TGF-β and Bone Morphogenetic Protein 4 (BMP4) inhibit VSMC proliferation and migration while inducing contractile gene expression.^19,37−39^ Serum deprivation enhances contractile gene expression, which can be reversed upon restoring a serum-rich medium, resulting in decreased contractile gene expression and a morphological transition of VSMCs. Notably, serum and PDGFB deprivation of human pluripotent stem-cell (hPSC)-derived VSMCs have been observed to induce maturation toward a contractile phenotype,^14^ whereas the use of a high-serum medium in conjunction with PDGFB treatment has been found to induce the synthetic phenotype in these hPSCs.

Based on these previous reports, we used serum and PDGFB starvation along with TGF-β treatment in our experiments to assess the capacity of ODMCs to acquire a contractile phenotype. Our results demonstrated that ODMCs, like VSMCs, successfully acquired a contractile phenotype when exposed to the “contractile” culture conditions. This was evidenced by the upregulation of contractile markers, a reduction in cell proliferation rate, and elongation of cells as compared to the control conditions (ODMCs and VSMCs in 10% serum) and the synthetic conditions (ODMCs and VSMCs in 10% serum, with PDGFB and TGF-β). Stimulation with the synthetic culture medium did not elicit any differences in marker expression, cell proliferation rate, or morphology in ODMCs or VSMCs, compared to the control conditions. It has been observed that (prolonged) *in vitro* expansion of VSMCs can lead to a gradual loss of the contractile phenotype.^40^ The lack of response to the “synthetic” culture medium could indicate that the control conditions utilized in our experiments already maintained a more synthetic population of ODMCs and VSMCs. Nevertheless, our findings demonstrate that ODMCs exhibit a level of phenotype plasticity that is comparable to that of VSMCs.

### 4.2 Higher Young’s Modulus and Cross-Linking Density in a Static 3D GelMa Environment Promotes a Contractile Phenotype in ODMCs and VSMCs

Based on gelatin modified with methacryloyl groups, GelMa is biocompatible and biodegradable and is widely used in various tissue engineering strategies, including 3D cell printing to recapitulate blood vessels or vascularized tissues.^41^ The mechanical properties of GelMa are tunable by altering its cross-linking conditions, including polymer concentration, degree of methacrylation, light wavelength and intensity, and light exposure time.^42,43^ The viability, function, and survival of GelMa-loaded cells are highly dependent on the resulting cross-linking density. Here, we used two different degrees of methacrylation (or degree of functionalization, DOF), 50 and 80%. Of these two DOFs, we used two different hydrogel weight percentages, 5 and 10%, to create four different hydrogels, each with distinct intrinsic matrix properties: (1) High cross-linking density, high Young’s modulus (80 DOF in 10% hydrogel), (2) high cross-linking density, low Young’s modulus (80 DOF in 5% hydrogel), (3) low cross-linking density, high Young’s modulus (50 DOF in 10% hydrogel), (4) low cross-linking density, low Young’s modulus (50 DOF in 5% hydrogel). For vascular cells, high cross-linking density in GelMa was previously shown to be detrimental to vascular network formation *in vitro* and *in vivo*, resulting in less and shorter neovessels with fewer branchpoints.^44,45^ Although the impact of a high degree of GelMa cross-linking on VSMCs was not investigated, the mesenchymal stem cells that were used for vascular support in these studies showed significant reduction in perivascular recruitment by neovessels and *in situ* impairment of differentiation into mural cells. A higher degree of cross-linking has also been associated with reduced cell spreading capacity by increasing the physical matrix barrier and reduction in pore size.^46^ In line with these observations, ODMCs and VSMCs in 80 DOF in 10% hydrogels showed limited elongation in cell morphology compared to cells in 50 DOF (in 10 and 5%) or 80 DOF in 5% hydrogel under static conditions, indicative of impairment in cell spreading. Dynamic stimulation of the 80 DOF in 10% hydrogel condition only induced limited morphological adaptation in VSMCs but not ODMCs, and strained VSMCs displayed nontypical cell thinning or enlargement instead of elongation (Figure S3). A higher degree of cross-linking in GelMa was previously reported to reduce expression of mural cell markers in mesenchymal stem cells,^44^ but the impact on VSMC phenotype switching in hiPSC-derived mural cells remained to be investigated. Here we observed under static conditions a significantly higher expression of contractile markers in 80 DOF versus 50 DOF, in 5% and to a lesser extent in 10% hydrogels, with ODMCs performing better than VSMCs (Figure 4A,B), demonstrating that higher cross-linking density of 3D hydrogels promotes a contractile phenotype.

Phenotype determination in primary VSMCs may also be controlled by the elastic modulus of the hydrogels. The limited data available on the effect of these parameters on VSMC behavior are derived from 2D experiments. 2D studies have also highlighted the impact of different ECM components. For example, Collagen type I coating on substrates with an increasingly higher Young’s modulus reduced (synthetic phenotype associated) VSMC migration, whereas under similar conditions, a fibronectin coating promoted migratory behavior.^24^ Notably, it has been indicated that the migratory response to substrates within a range of 1.0–308 kPa (Young’s modulus) is biphasic, implying that there is an optimum for maximal migration.^25^ Another interesting observation is that substrates with a higher Young’s modulus require a lower density of ECM (fibronectin) coating to achieve a similar migratory response in VSMCs than substrates with a lower modulus.^25^ These findings indicate that the response of VSMCs to the mechanical substrate properties in 2D is highly dependent on the assessed Young’s modulus range and the ECM component type and density. How these findings will translate in a more physiologically relevant 3D environment, in particular, for hiPSC-derived VSMCs, remains largely underexplored. A recent comparative investigation focusing on the cyclic stretching stimulation of human VSMCs showed contrasting outcomes between 2D and 3D models, with contractile protein expression remaining unaltered under 2D stretching conditions, and exhibiting a notable increase within 3D collagen matrix conditions.^28^ These disparities underscore the possible critical influence of extracellular dimensionality (2D or 3D) on cellular responses to mechanical stimulation, emphasizing the urgent need to broaden the scope of current investigations in this domain. By comparing 5% with 10% hydrogels, our data showed that 3D GelMa hydrogels with a higher Young’s modulus in a static environment increased the expression of contractile markers in ODMCs under both 80 and 50 DOF conditions. The same effect was observed to a lesser extent for VSMCs for 80 DOF hydrogels (Figures S1C and 4B). These results are partially in line with the previous findings. Peyton et al. showed that adjusting the modulus within the range of 0.45–5.8 kPa resulted in the modulation of cytoskeletal assembly in human primary VSMCs in a 3D PEG-fibrinogen based static hydrogel, with stiff matrices exhibiting a slightly elevated level of F-actin bundling.^47^ However, the expression of contractile markers in Peyton’s study was increased only in matrices with a higher Young’s modulus after constitutive RhoA activation, which may be attributed to the use of different hydrogels (GelMa versus PEG-fibrinogen). Similar to Peyton’s findings, static 3D culture with different matrix properties (5 versus 10% hydrogels) had no effect on the cell survival of ODMCs and VSMCs. Cross-linking density also had no impact on the survival of ODMCs (Figure 3D). Combined, these findings demonstrate, for the first time, that increasing the Young’s modulus in GelMa-based hydrogels promotes a viable contractile phenotype in hiPSC-derived VSMCs.

Increasing the strength of GelMa as a bio-ink for 3D printing may not only offer mechanical stability to aid during the fabrication but also ultimately create vessel grafts with higher vessel wall strength to better withstand physiological flow ranges. A new dual cross-linking method was recently reported for vascular 3D printing, which combines photo-cross-linking with enzymatic cross-linking facilitated by glucose peroxidase and horseradish peroxidase, which resulted in a construct with higher substrate strength.^48^ However, the biological performance was investigated with endothelial cells seeded on top of the gel in the lumen of the channels created by sacrificial printing. Although this showed adequate cell adhesion and viability for the endothelium, similar parameters were not tested in a condition in which vascular (mural) cells were suspended in the dual cross-linked gel in 3D.^48^

### 4.3 Uniaxial Strain Induces a Phenotypic Switch toward a Contractile Population of Smooth Muscle Cells in ODMCs and VSMCs

The effect of cyclic strain on the morphology and function of VSMCs has been described predominantly in 2D setups. The use of cyclic strain in 2D within the pathologically relevant range (>15%) has been shown to induce DNA synthesis through increased reactive oxygen species (ROS) production and NF-kB pathway activation.^49^ Conversely, physiological strain levels (10%) inhibit VSMC proliferation by upregulating p21 expression and promoting apoptosis.^50,51^ For human iPSC-derived VSMCs, data on cyclic strain is very limited, with findings that indicate an ability for cytoskeletal remodeling in response to 2D strain similar to primary VSMCs in a progeria-on-a-chip model.^52^ In relation to the expression of phenotypic markers, up- and downregulation of contractile markers have been reported following VSMC exposure to cyclic strain.^53−57^ Most notably, Bono et al. compared the impact of cyclic strain on VSMCs cultured in type I Collagen substrate in both 2D and 3D environments.^28^ In the 2D model, they reported a downregulation of contractile proteins (αSMA and Calponin) in the strained versus static samples. In contrast, a 2-fold increase in αSMA and 14-fold increase in Calponin expression was observed in the 3D conditions when exposed to cyclic stain. This coincided with a difference in morphological adaptation with a perpendicular (80–90°) alignment in the 2D cultured versus parallel (0–10°) alignment in 3D cultured VSMCs, in relation to the strain direction. These findings imply that VSMC adaptation to cyclic strain is profoundly different in 2D versus 3D conditions. In line with these 3D findings from Bono’s study, we observed that cyclic strain significantly increased the expression of contractile markers and caused elongation of ODMCs and VSMCs in the direction of the applied strain in all four of our 3D hydrogel conditions. Cross-linking density (comparing the 80 and 50 DOF hydrogels) and matrix Young’s modulus (comparing 10 and 5% hydrogels) did not affect this contractile switch in both cell types. Strain pattern comparison of the 80 DOF in 10% versus the 50 DOF in 5% hydrogels did not show any significant differences, indicating that variations within this range may have limited impact on the local strain levels of what the cells experience on an individual level. Future research should investigate hydrogels with a higher range in cross-linking density and elastic modulus to assess the impact of hydrogel properties on the conveyance of strain from the tissue to the cellular level.

## 5 Conclusions

In this study, we demonstrated the phenotypic plasticity of hiPSC-derived ODMCs, which have the capacity to adopt a contractile phenotype in response to growth factor stimulation in 2D. In addition, 3D culture in GelMa hydrogels under static conditions showed that properties like a higher Young’s modulus and higher cross-linking density induced a contractile phenotype in these cells, similar to VSMCs. Dynamic stimulation in the 3D substrate using uniaxial strain further promoted a switch toward a contractile phenotype in both ODMCs and primary VSMCs.

These findings underscore the significance of optimizing matrix properties within a (dynamic) 3D environment and contribute to the advancement of sophisticated human disease models and vascular tissue engineering strategies.

## Supporting Information Available

Comparative gene expression analysis in different DOFs; gene expression analysis of the effect of growth factors in 3D; immunofluorescent hydrogel staining; strain analysis without cells; and immunofluorescent staining of strained gels (PDF)
