# Supplementary material for: Effect of Mechanical Stimuli on the Phenotypic Plasticity of Induced Pluripotent Stem-Cell-Derived Vascular Smooth Muscle Cells in a 3D Hydrogel
Source: ACS Appl Bio Mater. 2023 Nov 30;6(12):5716–29. doi: 10.1021/acsabm.3c00840 (PMC10731661; doi:10.1021/acsabm.3c00840)
Supplement: Supplementary file 1 — mt3c00840_si_005.pdf [file mt3c00840_si_005.pdf]

Supporting Information

The Effect of Mechanical Stimuli on the Phenotypic Plasticity of Induced Pluripotent Stem Cell-Derived Vascular Smooth Muscle Cells in a 3D Hydrogel

Elana M. Meijer[1,2#], Rachel Giles[1,2#] Christian G.M. van Dijk[1,2~], Ranganath Maringanti[1,2,3~], Tamar B. Wissing[4,5], Ymke Appels[1,2], Ihsan Chrifi[1,2,3], Hanneke Crielaard[6], Marianne C. Verhaar[1,2], Anthal I.P.M. Smits[4,5], Caroline Cheng[1,2,3*].

[1]Department of Nephrology and Hypertension, Division of Internal Medicine and Dermatology, University Medical Center Utrecht, Utrecht, The Netherlands; [2]Regenerative Medicine Center Utrecht, University Medical Center Utrecht, Utrecht The Netherlands; [3]Experimental Cardiology, Department of Cardiology, Thorax Center Erasmus University Medical Center, Rotterdam, The Netherlands; [4]Department of Biomedical Engineering, Eindhoven University of Technology; Eindhoven, The Netherlands ; [5]Institute for Complex Molecular Systems (ICMS), Eindhoven University of Technology; Eindhoven. [6] Department of Biomedical Engineering, Erasmus Medical Center, Rotterdam, the Netherlands.

[#,~]Contributed equally

*Corresponding author:

Caroline Cheng, PhD

University Medical Center Utrecht

PO Box 85500, 3508 GA, Utrecht, The Netherlands

T: +31 (0)-88-7557329

E-mail: K.L.Cheng-2@umcutrecht.nl

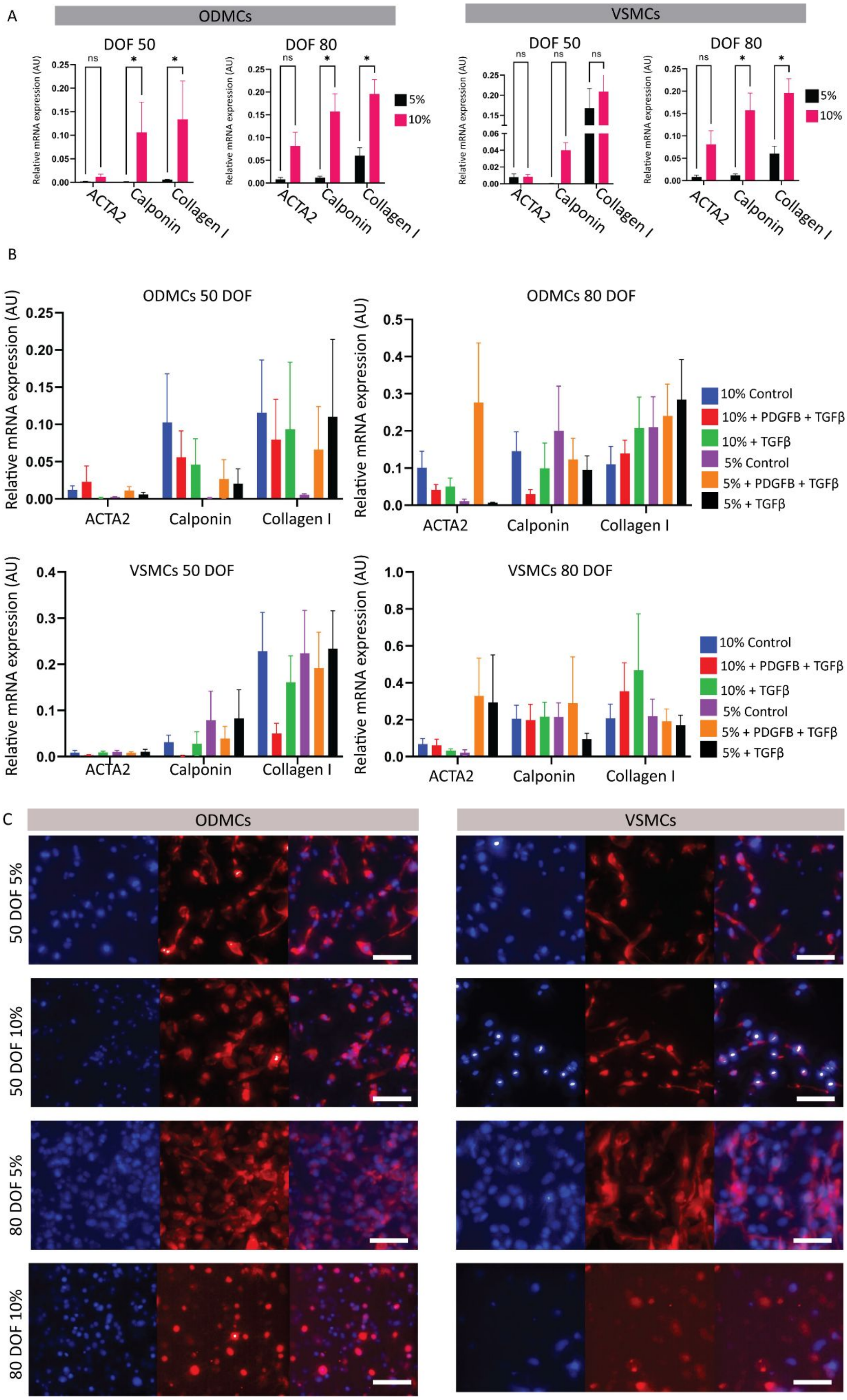
A
ODMCs
VSMCs
DOF 50
DOF 80
Relative mRNA expression (AU)
ns
*
5%
10%
ACTA2
Calponin
Collagen I
B
ODMCs 50 DOF
ODMCs 80 DOF
VSMCs 50 DOF
VSMCs 80 DOF
10% Control
10% + PDGFB + TGFβ
10% + TGFβ
5% Control
5% + PDGFB + TGFβ
5% + TGFβ
C
50 DOF 5%
50 DOF 10%
80 DOF 5%
80 DOF 10%

Supplemental figure S1. Static 3D GelMa experiments. A. *Gene expression analysis of the ODMCs and VSMCs 48 h after seeding. Data represented as mean ± SEM, n=6 for ODMCs, n=5 for VSMCs. One-way ANOVA with Tukey post hoc test, *p<0.05.* ***B.*** *Gene expression analysis of the ODMCs and VSMCs 48 h after seeding. Data represented as mean ± SEM, n=3 for both conditions. One-way ANOVA with Tukey post hoc test, no significant differences in relative mRNA expression was observed.* ***C.*** *Immunofluorescent whole mount staining of ACTA2 (red). Stained GelMa gels 48 h after seeding. DAPI (blue) was used as a counterstain. Scale bar depicts 50µm.*

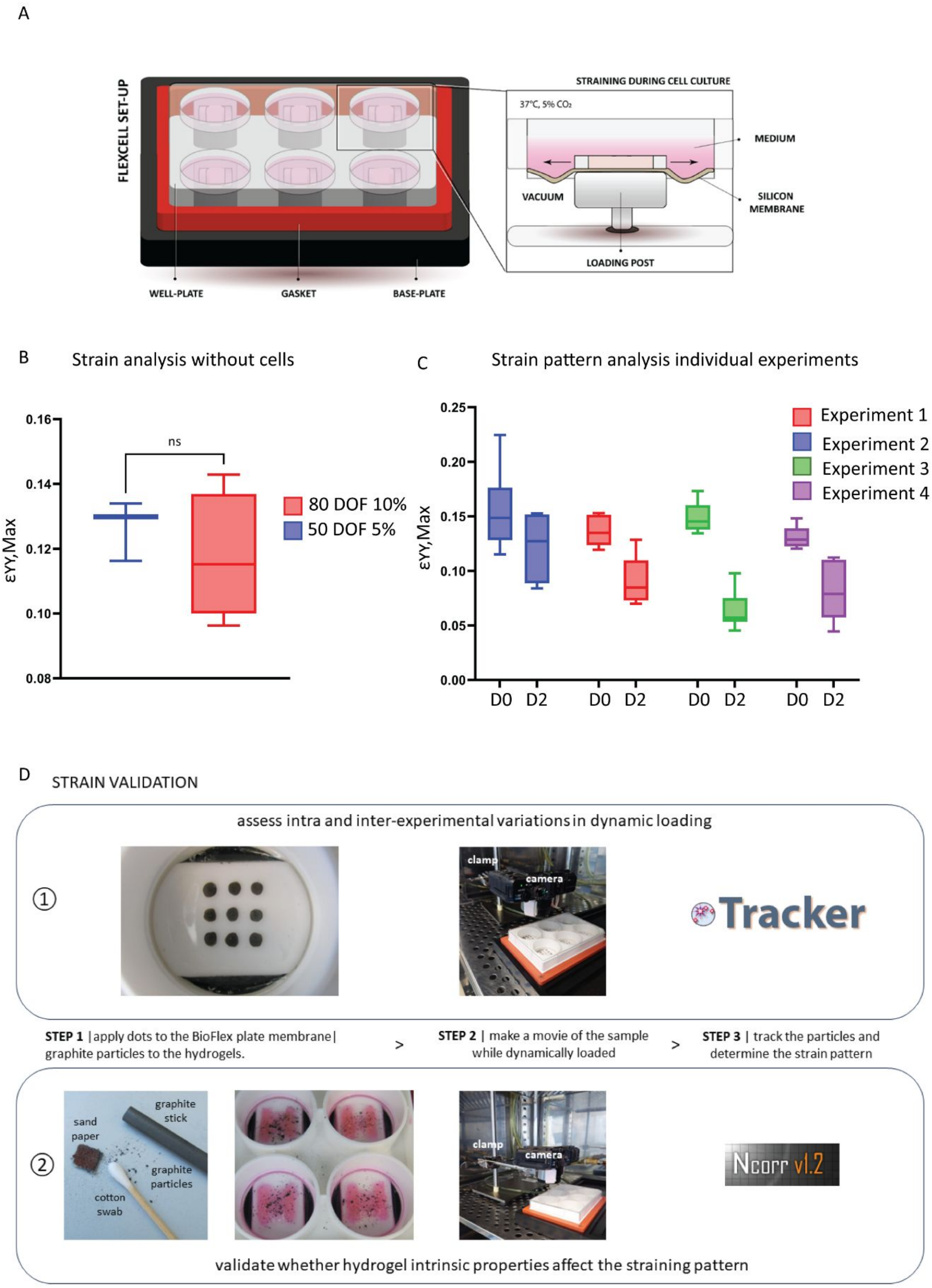

Supplemental figure S2. Strain analysis with and without gels. A. *Graphical representation of the Flexcell set-up that was used to dynamically load the hydrogels. The BioFlex culture plate containing the hydrogels is placed in the baseplate of the Flexcell system and sealed with a gasket to maintain the vacuum that is applied underneath the plate. This vacuum will induce a stretch in the silicon membrane of the Flexcell plat and the hydrogel that is connected to this membrane. The shape of the loading post*

*will control the directionality of the stretch. In this experiment arctangle loading posts were used to apply a unidirectional stretch***. B.** *Strain analysis of both 80 DOF 10% and 50 DOF 5% gels 72 h after crosslinking. Data represented as maximum strain, n=3 individual experiments. Data represented as Min-Max, unpaired t-test detected no significant differences.* ***C.*** *Strain analysis of dotted, unseeded FlexCell© plates without gels to compare strain levels between different experiments. n=6 dots measured per experiment. Data is represented as Min-Max, Two-way ANOVA detected no differences between day 0 and day 2 and between individual experiments.* ***D.*** *Clarification of the two strain validation approaches that we conducted during the experiment. Method ① was used to asses intra- and inter-experimental variation in straining while method ② was used to assess the straining pattern of the hydrogels. Assessment of the straining pattern didn't show inhomogeneities due to potential slippage between the membrane of the BioFlex culture plate and the GelMA hydrogel.*

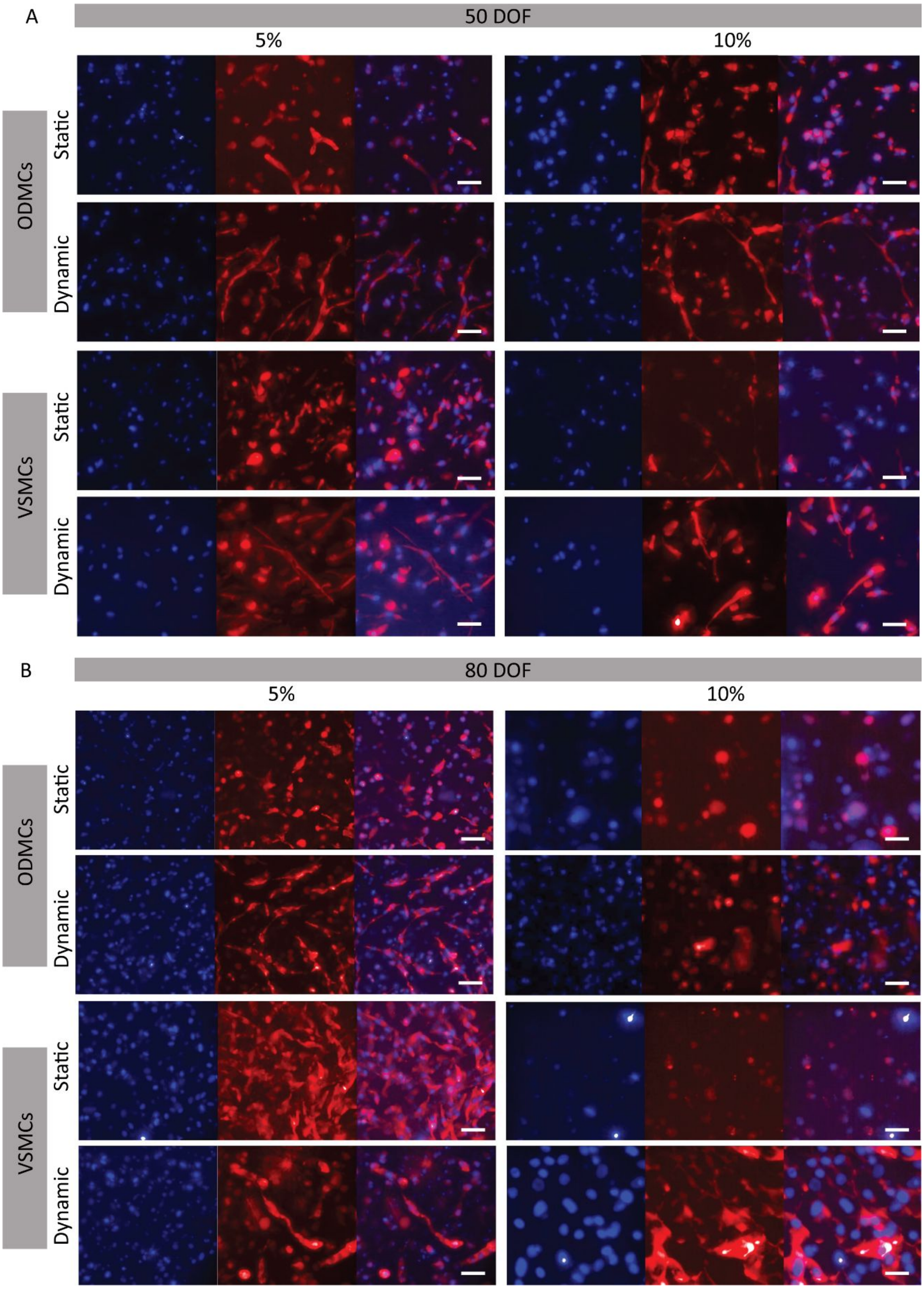

Supplemental figure S3. The effect of uniaxial cyclic strain on ODMCs and VSMCs in GelMa. A. *Immunofluorescent whole mount staining of ACTA2 (red). Stained 50 DOF GelMa gels after 48 h of 10% strain. DAPI (blue) was used as a counterstain. Scale bar depicts 50µm.* ***B.*** *Immunofluorescent whole mount staining of ACTA2 (red). Stained 80 DOF GelMa gels after 48 h of 10% strain. DAPI (blue) was used as a counterstain. Scale bar depicts 50µm.*

Supplemental table S1. FACs antibodies

Antibody	Conjugate	Catalog nr.	Company	Dilution
CD140b (Mouse anti-Human)	PE	558821	BD biosciences	1:25
CD31 (Mouse anti-Human)	Alexa Fluor 647	558094	BD biosciences	1:50
IgG1 Isotype Control Alexa Fluor 647	-	557783	BD biosciences	1:50
IgG Isotype control PE	-	IC003P	R&D Systems	1:25

Supplemental table S2. QPCR primer sequences

Gene	Forward primer	Reverse primer
ACTA2	ACTGGGACGACATGGAAAAG	GCGTCCAGAGGCATAGAGAG
Calponin	AGGCTCCGTGAAGAAGATCA	CTCCCACGTTCACCTTGTTT
Collagen 1a1	AAGACATCCCACCAATCACC	CGTCATCGCACAACACCTT
RPLP0 (housekeeping)	CCATTCTATCATCAACGGG	TCAGCAAGTGGGAAGGTGT

Supplemental table S3. Immunohistochemistry antibodies

Primary antibodies	Antigen	Antibody nr.	Company	Dilution
CD31	Mouse monoclonal	NB100-65900	DAKO	1:250
αSMA	Mouse monoclonal, CY3	CG198	Abcam	1:250
PDGFrβ	Goat polyclonal	AF358	R&D Systems	1:250
Calponin	Mouse monoclonal	C2687	Sigma	1:250
**Secondary antibodies**				
CD31	Donkey anti-mouse Alexa 568	A-10037	Invitrogen	1:500
PDGFrβ	Donkey anti-goat Alexa 488	A-11055	Invitrogen	1:500
Calponin	Goat anti-mouse Alexa 488	A-28175	Invitrogen	1:500
Phalloidin	Alexa 594	A-12381	Thermofisher	1:100
DAPI	-	D-1306	Thermofisher	1: 5000
